# Supplementary material for: Access to medicines among the Brazilian population based on the 2019 National Health Survey
Source: PLoS One. 2023 Jan 20;18(1):e0280599. doi: 10.1371/journal.pone.0280599 (PMC9858017; doi:10.1371/journal.pone.0280599)
Supplement: Attachment — Submitted filename: Response to Reviewers.docx [file pone.0280599.s001.docx]

Dear reviewers,

I am so grateful for the opportunity to review this manuscript and below I send the changes that were made:

| Reviewer #1 | |
| --- | --- |
| Reviewer's considerations | Authors' changes |
| In the Introduction section, we missed a slightly broader description of the framework in which health care is provided in Brazil, the organisation of its health care system, who is covered and how drugs are paid for at these different levels. Some of the considerations in the Discussion section would be better understood with this background information. | Included in Introduction:  “In Brazil, the health care model, including pharmaceutical care and access to medication, is made up of three systems: the universal public system, Unified Health System (SUS), with completely free access; the supplementary health system, which are private plans and insurance; and the “out of pocket” system, in which the service provider is paid directly [2].”  The second reference has been changed. |
| In the Methods section, the description of the dependent variable needs to be rewritten. Why is it stated that only affirmative answers to the question were considered? It would be valuable to attach information (maybe an annex) with the specific PNS questions that led to the definition of the variables. | It was rewritten:  We included responses of individuals who sought medical care in the last 15 days but only got it in the previous attempt (question J021 - "The last time you sought health care for this reason, in the last two weeks, was ___ seen?") (40,357 individuals), and answered “yes” regarding having received a medicine prescription (question J29a - "Was any medicine prescribed in this consultation for ___?") (24,753 responders). |
| Since it is noted that the descriptive analysis considered the sample weight and the effect of clustering, the authors should show which method was used to estimate the confidence intervals. In this section one would also expect to find information on how the best model was chosen and whether any measures of fit were assessed. | Was added in Statistics:  The analysis of the fit of the final model was performed using the likelihood ratio test and Pseudo R2. |
| The results section needs additional information. What does column n in table 1 mean? | Subtitles have been added. |
| In explaining the results of the multivariate analysis, it should be noted that the excess risk needs to be interpreted in relation to the reference category (having full access to prescribed medicines). This may seem obvious to the authors, but will not be so for readers unfamiliar with multinomial logistic regression. | It was rewritten:  Table 3 presents the adjusted analysis of access to prescription medicines in the Brazilian population using multinomial logistic regression, considering the category access to all prescribed drugs of the dependent variable as the analysis reference. |
| We believe that there must be an error as the risk of being in these categories is higher for women (table 3). This error is also shown in the results of the abstract. | The error has been corrected in the summary and results. |
| We do not know whether subjective reasons for lack of access to medication were asked. In the discussion section, the different reasons are considered from a theoretical point of view. If this was not done, it should be presented as a limitation of the study. Another type of limitation is associated with the study design (cross-sectional). Associations can be studied, but causality cannot be attributed to these associations. Therefore, the first conclusion cannot be derived from the results of the study. The way the conclusions are presented in the abstract seems more appropriate. | Study limitations were clarified, and the beginning of the conclusion text was adjusted. |
| Reviewer #2 | |
| A recently published article analysed data from the same data source (PNS) comparing 2013 and 2019, coinciding with the time frame of present manuscript, but no discussion is provided on how the data compare and if there are significant differences in the results. | Was added:  Specifically, Boing and other authors observed that the proportion of people who did not obtain no medication in the SUS and that made some direct disbursement increased, comparing the results of the PNS between 2013 and 2019, and that the probability of obtaining all the medications in the SUS was higher among the poorest [31]. |

The authors believe that they have responded to all requests for review and are awaiting a new opinion from Plos One.

Forward to your response.

Yours sincerely,

Adriana Amorim de Farias Leal
